# Non-invasive evaluation of neuroprotective drug candidates for cerebral infarction by PET imaging of mitochondrial complex-I activity

**DOI:** 10.1038/srep30127

**Published:** 2016-07-21

**Authors:** Tatsuya Fukuta, Tomohiro Asai, Takayuki Ishii, Hiroyuki Koide, Chiaki Kiyokawa, Masahiro Hashimoto, Takashi Kikuchi, Kosuke Shimizu, Norihiro Harada, Hideo Tsukada, Naoto Oku

**Affiliations:** 1 Department of Medical Biochemistry, School of Pharmaceutical Sciences, University of Shizuoka, 52-1 Yada, Suruga-ku, Shizuoka 422-8526, Japan; 2 Japan Society of the Promotion of Science (JSPS), 5-3-1 Kojimachi, Chiyoda-ku, Tokyo 102-0083, Japan; 3 PET Center, Central Research Laboratory, Hamamatsu Photonics K.K., Hirakuchi, Hamakita-ku, Hamamatsu, Shizuoka 434-8601, Japan

## Abstract

The development of a diagnostic technology that can accurately determine the pathological progression of ischemic stroke and evaluate the therapeutic effects of cerebroprotective agents has been desired. We previously developed a novel PET probe, 2-*tert*-butyl-4-chloro-5-{6-[2-(2-^18^F-fluoroethoxy)-ethoxy]-pyridin-3-ylmethoxy}-2H-pyridazin-3-one ([^18^F]BCPP-EF) for detecting activity of mitochondrial complex I (MC-I). This probe was shown to visualize neuronal damage in the living brain of rodent and primate models of neurodegenerative diseases. In the present study, [^18^F]BCPP-EF was applied to evaluate the therapeutic effects of a neuroprotectant, liposomal FK506 (FK506-liposomes), on cerebral ischemia/reperfusion (I/R) injury in transient middle cerebral artery occlusion rats. The PET imaging using [^18^F]BCPP-EF showed a prominent reduction in the MC-I activity in the ischemic brain hemisphere. Treatment with FK506-liposomes remarkably increased the uptake of [^18^F]BCPP-EF in the ischemic side corresponding to the improvement of blood flow disorders and motor function deficits throughout the 7 days after I/R. Additionally, the PET scan could diagnose the extent of the brain damage accurately and showed the neuroprotective effect of FK506-liposomes at Day 7, at which 2, 3, 5-triphenyltetrazolium chloride staining couldn’t visualize them. Our study demonstrated that the PET technology using [^18^F]BCPP-EF has a potent capacity to evaluate the therapeutic effect of drug candidates in living brain.

Ischemic stroke is the leading cause of long-term disability in adults worldwide^1^. Cerebral ischemia-reperfusion (I/R) injury, which often results from thrombolytic therapy, is strongly related to the disability in stroke patients and remains an important clinical problem^2^. To develop cerebroprotective agents for this injury, researchers have designed various drug candidates over the years. However, effective therapeutic agents and methods for this injury have remained to be established^3^,^4^. Thus, further research is required to develop a novel therapy for improving stroke outcome. In addition, advanced evaluation methods are required to precisely assess the therapeutic effect of candidate drugs on ischemic brain damage.

Positron-emission tomography (PET) is often used as a potent instrument for diagnosis of diseases and evaluating the therapeutic efficacy of drugs^5^,^6^. For evaluating the ischemic brain damage after an ischemic insult, we previously designed a PET probe, 2-*tert*-butyl-4-chloro-5-{6-[2-(2-^18^F-fluoroethoxy)-ethoxy]pyridin-3-ylmethoxy}-2H-pyridazin-3-one ([^18^F]-BCPP-EF)^7^, and evaluated its capability to detect neurodegenerative damage in the living rat brain^8^. [^18^F]-BCPP-EF is specific for detecting the activity of mitochondrial complex I (MC-I; NADH-ubiquinone oxidoreductase), which is the first enzyme of the electron-transport chain and involves in oxidative phosphorylation. Earlier, [^18^F]-fluorodeoxyglucose (FDG), a conventional PET probe, was applied for evaluating ischemic brain damage; however, PET scanning using [^18^F]FDG showed unexpectedly high signals caused by inflammation resulting from microglial activation at the subacute phase after an ischemic stroke^8^,^9^,^10^. [^18^F]BCPP-EF was shown to provide more reliable images of ischemic brain damage than other existing methods without interference by inflammatory reactions^8^,^10^. However, the potential of [^18^F]BCPP-EF for therapeutic evaluation has not been elucidated at all. Therefore, we evaluated the neuroprotective effects of drug candidates by PET scanning with [^18^F]BCPP-EF in the present study.

As a neuroprotectant, we used liposomal FK506 (FK506-liposomes) in this study. FK506 (tacrolimus) is used as an immunosuppressant in a number of countries, and is known to possess neuroprotective efficacy due to its ability to inhibit calcineurin activity^11^,^12^. Clinical trials of FK506 for ischemic stroke therapy were conducted in the US; but, unfortunately, they were terminated due to undesirable side effects. Our previous study revealed that liposomalization of FK506 enhances its neuroprotective effect against cerebral I/R injury in transient middle cerebral artery occlusion (t-MCAO) rats, which procedure could lead to decreased rates of side effects by allowing a reduction in the dose of the drug^13^. Therefore, FK506-liposomes would be useful for evaluating [^18^F]BCPP-EF as a PET probe to visualize their cerebroprotective effect.

In the present study, we examined the therapeutic effect of FK506-liposomes on cerebral I/R injury in t-MCAO rats at both acute and subacute phases, by chronologically assessing the MC-I activity by PET analysis using [^18^F]BCPP-EF, the motor functional behavior, and the cerebral blood flow. Through these analyses, we investigated the potential of [^18^F]BCPP-EF as a novel diagnostic probe for non-invasively evaluating the neuroprotective effect of drug candidates on cerebral injury.

## Results

### Amelioration of motor function deficits by the treatment with FK506-liposomes

**Figure 1:**
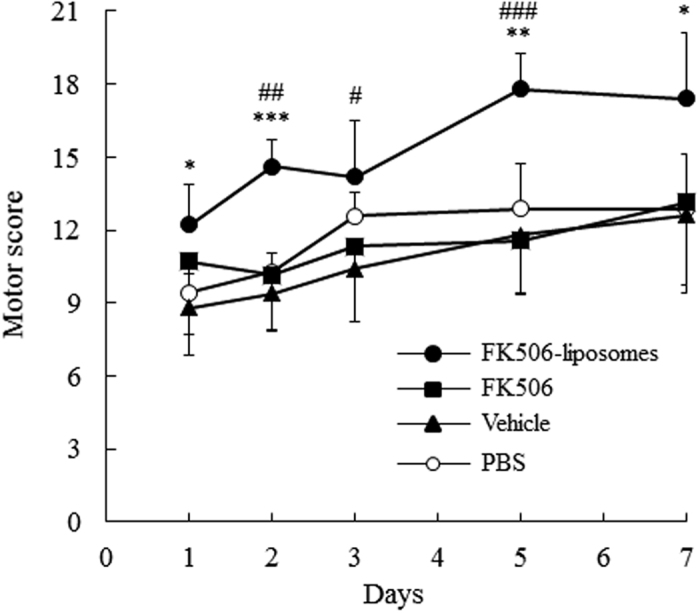
Amelioration of motor function disorder of t-MCAO rats by treatment with FK506-liposomes. t-MCAO rats were intravenously injected with PBS, vehicle, FK506 (100 μg/kg) or FK506-liposomes (100 μg/kg FK506 dose) immediately after reperfusion following a 1-h occlusion. At 1, 2, 3, 5 and 7 days after reperfusion, the motor function of the t-MCAO rats was evaluated according to the 21-point neuropathological score. Data are presented as the mean ± S.D. (n = 7 for PBS, FK506, and FK506-liposomes groups, and n = 5 for vehicle group). The significance of differences is indicated as follows: *P < 0.05, **P < 0.01, ***P < 0.001 *vs*. PBS, ^#^*P* < 0.05, ^##^*P* < 0.1 ^###^*P* < 0.001 *vs*. FK506.

The particle size and ζ-potential of FK506-liposomes were 106.5 ± 1.7 nm and -2.62 ± 1.1 mV, respectively. The encapsulation efficiency of FK506 into liposomes was 53.3 ± 12.3%. We previously reported that 100-nm liposomes can pass through a disintegrated blood-brain barrier and accumulate in the I/R region of t-MCAO rats from an early phase of reperfusion^14^. At first, the therapeutic effect of FK506-liposomes (100 μg/kg FK506 equivalent, 2 μmol phospholipid/rat) on neurological disorders was examined by monitoring motor functions of t-MCAO rats at 1, 2, 3, 5, and 7 days after reperfusion. Our previous study revealed that an intravenous administration of FK506-liposomes (100 μg/kg FK506 equivalent) is effective for the treatment of the acute phase of cerebral I/R injury^13^. As a result, the treatment with FK506-liposomes significantly improved the motor functions of the t-MCAO rats compared with that with the same dose of FK506 or PBS from 1 day of reperfusion (Fig. 1). Moreover, the improvement was also observed at day 7. In particular, motor functional deficits were markedly ameliorated between day 3 and day 5 in the FK506-liposome-treated group. Similar to previous findings^15^, we confirmed that the vehicle had no therapeutic effect on the rats.

### Improvement of secondary blood flow disorder by intravenously injected FK506-liposomes

**Figure 2:**
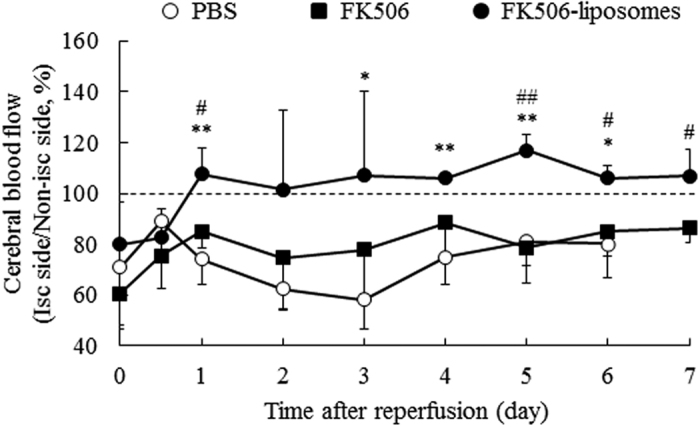
Cerebral blood perfusion deficit was improved by the treatment with FK506-liposomes. t-MCAO rats were injected with PBS, FK506 (100 μg/kg) or FK506-liposomes (100 μg/kg FK506 dose) via a tail vein. Cerebral blood flow of the t-MCAO rats was chronologically measured with a laser Doppler blood flowmeter at 0, 0.5, 1, 2, 3, 4, 5, 6, and 7 days of reperfusion. The graph indicates the ratio of the blood flow in the ischemic side to that in the non-ischemic side. The data show the mean ± S.D. (n = 4). Significance of differences: *P < 0.05, **P < 0.01 *vs*. PBS, ^#^*P* < 0.05, ^##^*P* < 0.01 *vs*. FK506.

Next, the cerebral blood flow of t-MCAO rats was examined chronologically with a laser Doppler flowmeter. In the control group, the reduction in cerebral blood flow, which is thought to result from a secondary blood circulation disorder, was clearly observed (Fig. 2). In contrast, the disorder was significantly improved by the treatment with FK506-liposomes compared with the control and FK506-treated group. Additionally, the improvement of the cerebral perfusion deficits persisted for up to 7 days after reperfusion. Contrary to the viability in the FK506-liposome-treated group, half of the rats in the PBS-treated group died before 6 days of reperfusion in this experiment.

### Therapeutic effect of FK506-liposomes on brain cell damage induced by I/R

**Figure 3:**
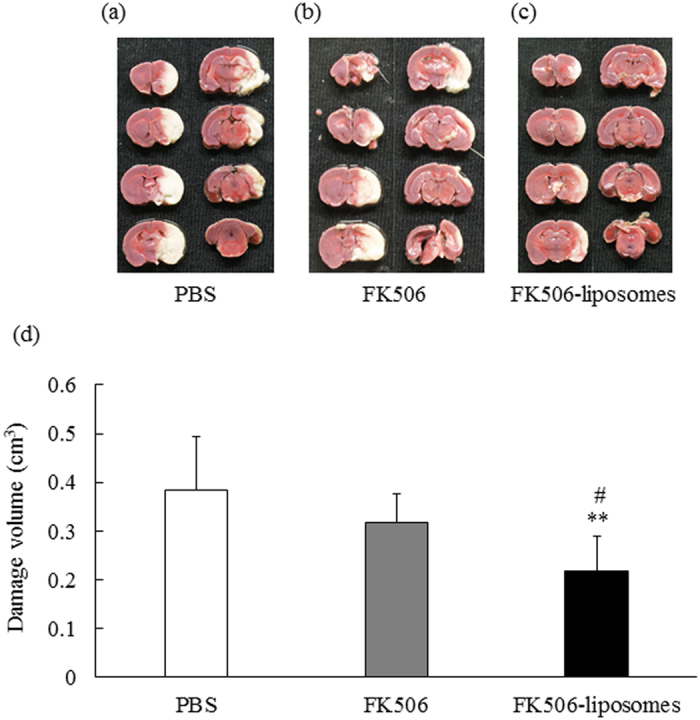
FK506-liposomes suppressed brain damage of t-MCAO rats at 3 days of reperfusion. PBS, FK506 (100 μg/kg) or FK506-liposomes (100 μg/kg FK506 dose) were intravenously injected into t-MCAO rats just after reperfusion. At 3 days of reperfusion, 2-mm coronal brain sections were prepared and stained with 2% TTC at 37 °C for 30 min (**a–c**). The damaged brain volume was assessed by Image J (**d**). Data are presented as the mean ± S.D. (n = 6 for PBS group, n = 7 for FK506 group, and n = 8 for FK506-liposomes group). Significance of differences: **P < 0.01 *vs*. PBS, ^#^*P* < 0.05 *vs*. FK506.

The cerebroprotective effect of FK506-liposomes on the t-MCAO rats was evaluated by TTC staining at 3 days after reperfusion. In the PBS- and FK506-treated groups, brain cell damage judged by TTC staining was extensive in the ischemic side (Fig. 3a,b,d). On the other hand, the administration of FK506-liposomes significantly suppressed the brain damage compared with that seen in the other groups (Fig. 3c,d). In particular, the damage around the striatum, which receives motor information from the motor area of the cortex, was remarkably reduced by the treatment with FK506-liposomes.

### Neuroprotective effect of FK506-liposomes against cerebral I/R injury

**Figure 4:**
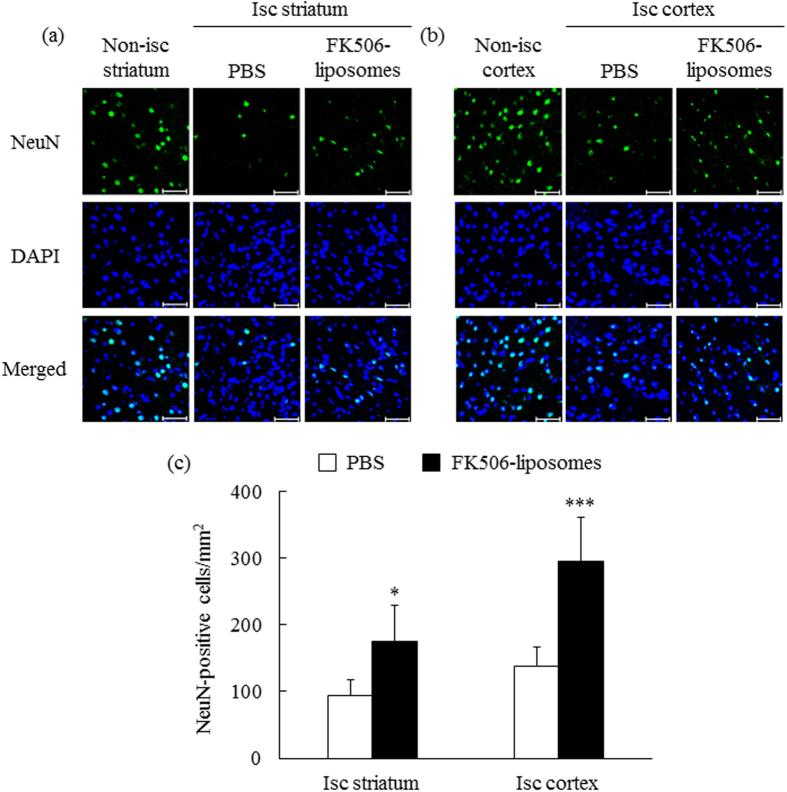
Neuroprotective effect of FK506-liposomes against I/R injury. t-MCAO rats were intravenously injected with FK506-liposomes (100 μg/kg FK506 equivalent) or PBS immediately after reperfusion. Seven days after reperfusion, 10-μm frozen brain sections were prepared and immunostained for NeuN (green) and counterstained nuclei with DAPI (blue). The fluorescence images of the striatum (**a**) and the cortex (**b**) were obtained by confocal laser scanning microscopy. Isc and non-isc indicate ischemic and non-ischemic areas, respectively. Scale bar, 50 μm. Quantitative data from confocal images were obtained by counting NeuN-positive cells in the sections (**c**). Data are presented as the mean ± S.D. (n = 5). Significance of differences: *P < 0.05, ***P < 0.001 *vs*. PBS.

Next, the neuroprotective effect of FK506-liposomes was immunofluorescently examined by observing neuronal nuclei (NeuN)-positive cells in the brains of t-MCAO rats 7 days after reperfusion. The confocal images showed that the number of NeuN-positive cells in both the striatum and the cortex was clearly reduced by I/R injury (Fig. 4a,b). However, the reduction of these cells was clearly ameliorated by the treatment of the rats with FK506-liposomes (Fig. 4a,b). The results of quantitative analysis demonstrated the significant neuroprotective effect of FK506-liposomes against I/R injury (Fig. 4c).

### Therapeutic evaluation of FK506-liposomes by PET using [^18^F]BCPP-EF

**Figure 5:**
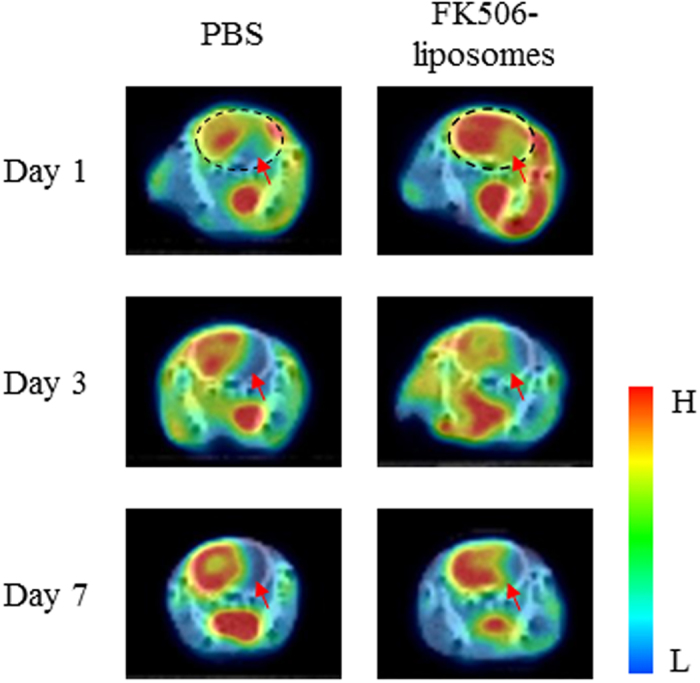
Evaluation of the neuroprotective effect of FK506-liposomes by use of the MC-I-specific PET probe [^18^F]BCPP-EF. PET scans were performed for 60 min with [^18^F]BCPP-EF (10 MBq/rat) at 1, 3, and 7 days after reperfusion. Summation PET images from 10 to 30 min were reconstructed to obtain standardized uptake value (SUV) images. The PET images were superimposed on the corresponding X-CT images. The black dotted circles (the images in day 1) indicate the position of the brain in the PET/CT images. The red arrows in the images indicate the ischemic hemisphere (right brain hemisphere), and the bar shows the relative level of signal intensity ranging from high (red) to low (blue).

**Figure 6:**
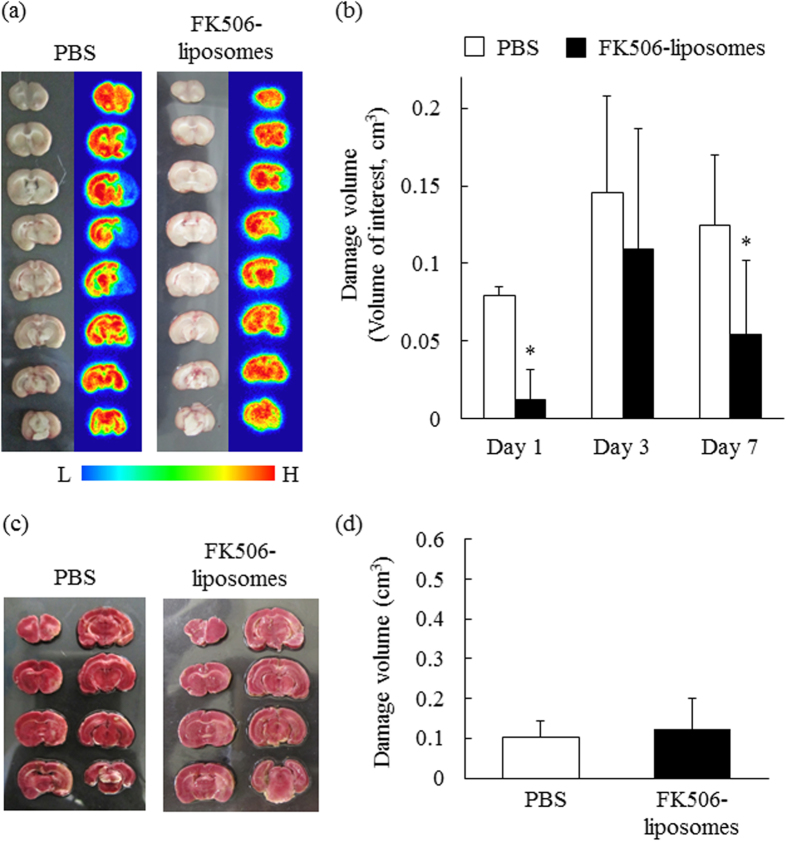
Visualization of the neuroprotective effect of FK506-liposomes by PET using [^18^F]BCPP-EF. PET scans were conducted for 60 min as described in the legend of Fig. 5. (**a**) Representative images of brains (a: left) and autoradiograms (a: right) after PET measurement at 7 days after reperfusion. The right brain hemisphere in the slices shows the ischemic hemisphere. The bar indicates the relative level of signal intensity, ranging from high (red) to low (blue). (**b**) The volume of brain damage was determined by obtaining VOIs in the reconstructed [^18^F]BCPP-EF PET images at day 1, 3 and 7. (**c,d**) The damaged brain volume assessed by TTC staining at day 7 was calculated using Image J. Data are presented as the mean ± S.D. (n = 5). Significance of differences: *P < 0.05 *vs*. PBS.

Finally, we performed PET analysis for evaluating MC-I activity after cerebral I/R and the effect of FK506-liposomes on the MC-I activity. As shown in the SUV images in Fig. 5, the uptake of [^18^F]BCPP-EF in the ischemic side was decreased in the PBS-treated group, especially at 3 and 7 days of reperfusion (Fig. 5), indicating that MC-I activity of the brain cells in the ischemic side had been impaired. In contrast, the treatment with FK506-liposomes remarkably increased the uptake of it at each day. Also, the autoradiograms obtained at day 7 showed increased [^18^F]BCPP-EF uptake in the ischemic side of the FK506-liposome-treated group (Fig. 6a). In addition, the results of volume of interest (VOI) analysis showed that the treatment with FK506-liposomes remarkably decreased damaged brain volume, especially at day 1 and 7 after I/R (Fig. 6b). These results well agreed with the improved motor function, and cerebral blood flow. After the autoradiography, the brain slices were stained with TTC. Of note, TTC staining showed no marked difference in the brain damage seen at Day 7 regardless of the treatment (Fig. 6c,d). Importantly, however, PET scanning with [^18^F]BCPP-EF successfully visualized the therapeutic response to FK506-liposomes (Figs 5 and 6a,b).

## Discussion

As one of the major problems for ischemic stroke patients, cerebral I/R injury, as exemplified by massive brain cell damage, often occurs after the recovery from brain ischemia. In the present study, we prepared a PET probe specific for MC-I activity to visualize the ischemic brain damage from the acute through subacute phase, and to evaluate the therapeutic effect of FK506-liposomes on cerebral I/R injury. It has been reported that unexpectedly high [^18^F]FDG uptake and apparent intense staining with TTC are observable in the damaged region at the subacute phase after cerebral I/R in spite of the neurodegenerative death in the area^16^. This phenomenon results from the exclusive production of ATP by activated inflammatory cells such as microglia and macrophages via enhanced glycolysis, the metabolic pathway with a low contribution of the electron transport chain for ATP production^17^,^18^. The advantage of [^18^F]BCPP-EF is its capability to precisely detect neuronal damage even at the subacute phase (Day 7) by specific binding to MC-I^8^,^10^. [^18^F]BCPP-EF is advantageous in terms of visualizing brain damage without killing the living animals in contrast to TTC. As shown in Fig. 6c,d, almost no difference was observed in the TTC-positive damaged brain area and between PBS- and FK506-liposome-treated groups at Day 7. However, the uptake of [^18^F]BCPP-EF was markedly lower in the ischemic side than in the non-ischemic side in the PBS-treated group; whereas the treatment with FK506-liposomes increased the uptake of it at each day (Figs 5 and 6). Moreover, the confocal images showed that the administration of FK506-liposomes significantly protected neuronal cells from I/R injury (Fig. 4). These results suggest that [^18^F]BCPP-EF, a probe with specific affinity for MC-I, could be a promising PET probe for evaluating ischemic neuronal damage at the subacute phase.

Assessment of motor performance is necessary to evaluate the efficacy of neuroprotective agents. Hence, the therapeutic effect of FK506-liposomes on motor function disorders was also evaluated in our present study. In the control, vehicle, and FK506-treated groups, the motor disorders were observed throughout the 7 days after reperfusion. However, intravenous administration of FK506-liposomes significantly improved them compared with the results for the other groups (Fig. 1). Notably, the amelioration was gradual, with improvement of hindlimb and forelimb hemiparesis, motility, and appearance; and it was remarkable between Days 3 and 5 of reperfusion. It was previously reported that cerebral I/R injury progresses at least until after 72 h of reperfusion due to post-ischemic inflammation^19^. As was shown in the results of Figs 3,5 and 6, this phenomenon was also marked in the control group. However, the results of the TTC study showed that FK506-liposomes significantly suppressed brain cell damage at 72 h after reperfusion compared with the control and the same dose used for the FK506-treated group (Fig. 3). Our previous study revealed that intravenously injected FK506-liposomes accumulated in the I/R region from an early phase after reperfusion, and markedly suppressed neutrophil infiltration and apoptosis around the striatum after 24 h of reperfusion following 1 h of occlusion^13^. It is well known that invasion of neutrophils into the brain parenchyma induces further ischemic damage due to their high content of proteolytic enzymes and reactive oxygen production, which cause additional progression of the inflammation^20^,^21^. Considering these previous findings, we propose that brain protection by FK506-liposomes from the acute phase contributed to the suppression of intracerebral post-ischemic inflammation, resulting in cerebroprotection and improvement of neurological deficits. In addition, as was shown in Figs 3 and 6, the areas protected by the treatment with FK506-liposomes were around the striatum which is one of the main components of the basal ganglia and has a key role in receiving motor information from the cerebral cortex. It was reported that the volume of brain damage in the striatum and cerebral cortex is well correlated with motor functional deficits in the MCAO rats^22^. It was also reported that mitochondrial complex dysfunction plays a crucial role in the progression of ischemic stroke^23^. From these findings, it is considered that the suppression of the MC-I impairment and of the damage around the striatum by the treatment with FK506-liposomes resulted in the amelioration of motor function deficits.

Microvascular dysfunction after cerebral I/R induces secondary blood flow disorders, resulting in a poor outcome for stroke patients. The therapeutic effect of FK506-liposomes on blood flow disorder was examined chronologically by using laser-Doppler flowmetry, and the results indicated that the treatment with FK506-liposomes significantly improved cerebral blood circulation (Fig. 2). It is well known that microcirculatory disorders are induced by the structural disruption of capillary vessels due to brain edema and that the obstruction caused by activated neutrophils and leukocytes results in the no-reflow phenomenon in the brain^24^,^25^,^26^. In light of these findings, we consider that the treatment with FK506-liposomes effectively suppressed brain edema and inflammation reactions around the brain tissue and cerebral vessels, resulting in the improvement of microcirculatory disorders. Moreover, the result of Fig. 2 well correlated with the reduction in the brain damage judged by PET analysis using [^18^F]BCPP-EF and by TTC staining, and with the amelioration of motor function. These results suggest that FK506-liposomes could be useful as a neuroprotectant for the treatment of cerebral I/R injury at both acute and subacute phases after an ischemic insult. Our previous study showed that the uptake of [^18^F]BCPP-EF was almost independent on regional cerebral blood flow in the monkey ischemic stroke model^10^. Therefore, it is also suggested that [^18^F]BCPP-EF could be a potent diagnostic tool for monitoring ischemic brain damage and the therapeutic benefits of neuroprotectants regardless of cerebral blood flow.

Since the only worldwide therapeutic drug option for the acute phase of ischemic stroke remains thrombolysis with recombinant tissue plasminogen activator, the development of a new effective therapeutic agent is to be desired^27^,^28^. For precise assessment of the neuroprotective efficacy of drug candidates toward ischemic brain damage, our present study revealed that the PET diagnosis of neurodegenerative damage using [^18^F]BCPP-EF has the capability of providing reliable information at both acute and subacute phases of a stroke. As the advantages of the therapeutic evaluation using PET, non-invasive and multiple diagnoses can be performed in the same living individuals. Based on these advantages, this technology has the potential to be applied to the drug discovery for some neurodegenerative diseases related to the MC-I activity such as ischemic stroke and Alzheimer’s disease. Our previous and present studies showed that [^18^F]BCPP-EF could be used to image impaired MC-I activity that correlated positively with ischemia-induced neuronal death and amyloid-β deposition in the brain of living rats and monkeys^10^,^29^. The specific binding of [^18^F]BCPP-EF to MC-1 was previously confirmed with rotenone, a specific MC-I inhibitor^7^,^8^. Furthermore, our current study is the first to show that PET scanning with [^18^F]BCPP-EF can be used to evaluate the therapeutic effect of neuroprotective agents on the neurodegenerative damage derived from I/R. Based on these findings, we expect that [^18^F]BCPP-EF will be used for not only detecting the progression of neurodegenerative diseases, but also therapeutic evaluation of drug candidates in rodents, primates, and humans. We also expect that this PET technology will accelerate the development of novel neuroprotectants worldwide.

In conclusion, the present study demonstrated that PET analysis using [^18^F]BCPP-EF as an MC-I-specific probe could detect the impaired MC-I activity induced by I/R, and also be used to evaluate the neuroprotective effect of FK506-liposomes in the living rat brain. Moreover, the results of PET scanning agreed with the therapeutic effect of FK506-liposomes on motor function deficits, secondary blood circulation disorder, and brain cell damage judged by TTC staining at day 3 in t-MCAO rats. From these results, we propose that [^18^F]BCPP-EF should be an useful tool for detecting neurodegenerative damage after an ischemic stroke as well as for evaluating the cerebroprotective effect of candidate neuroprotectants.

## Methods

### Animals

Eight-week-old male Wistar rats (180-210 g) were purchased from Japan SLC, Inc. (Shizuoka, Japan). All experimental protocols were approved by the Animal and Ethics Review Committee of the University of Shizuoka and the Central Research Laboratory, Hamamatsu Photonics., and all experiments were carried out in accordance with the Animal Facility Guidelines of the University of Shizuoka and the Central Research Laboratory, Hamamatsu Photonics.

### Preparation of FK506-liposomes

Dipalmitoylphosphatidylcholine (DPPC) and distearoylphosphatidylethanolamine (DSPE)-PEG2000 were gifts from Nippon Fine Chemical (Hyogo, Japan). FK506-liposomes composed of DPPC/DSPE-PEG2000 (20/1 molar ratio) were prepared by the freeze-drying method as described below. FK506 and lipid mixture in *tert*-butyl alcohol (DPPC/DSPE-PEG2000/FK506 = 20/1/0.4 as molar ratio) were added to an eggplant-shaped flask and lyophilized. Total lipid concentration of the liposomes was approximately 5 mM. The lyophilizate was hydrated with PBS (pH 7.4) at 50°C. After freeze-thawing with liquid nitrogen for 3 cycles, the liposomes were extruded through polycarbonate membrane filters having 100-nm pores (Nuclepore, Cambridge, MA, USA). Then, the particle size and ζ-potential of FK506-liposomes were measured using a Zetasizer Nano ZS (MALVERN, Worcestershire UK, USA). To remove unencapsulated FK506, the liposome solution was ultracentrifuged at 453,000 x *g* for 15 min (HITACHI, Tokyo, Japan). Then, the pellets were resuspended in PBS, and the FK506 concentration of the liposomes was determined by HPLC (HITACHI). FK506-liposomes were dissolved with tetrahydrofuran, and the solution was injected (20 μL) into an octadecylsilane column (TSK gel ODS-80TM, 4.6 × 150 nm, Tosoh, Tokyo, Japan). The mobile phase was composed of acetonitrile and water (3:2 v/v). The HPLC conditions were as follow: Column oven, 60 °C; flow rate, 1 mL/min; UV detection, 214 nm.

### Transient middle cerebral artery occlusion rats

t-MCAO rats were prepared as described previously^30^. Briefly, anesthesia was induced with 3% isoflurane (Escain^®^, Pfizer, NY, USA) and maintained with 1.5% isoflurane with a small-animal anesthesia apparatus (Model TK-4, Bio Machinery, Chiba, Japan). Rectal temperature was controlled at 37 °C with a heating pad (Unique Medical Co., Ltd., Tokyo, Japan). A midline ventral cervical skin incision was made, and then the right common carotid artery, external carotid artery, and internal carotid artery (ICA) were isolated. Next, a 4–0 silicon-coated nylon filament (18 mm; Keisei Medical Industrial Co., Ltd., Niigata, Japan) was inserted into the right ICA and gently advanced to the origin of the MCA. The neck incision was sutured, and the animal was allowed to recover from the anesthesia. The success of occlusion was judged by both the appearance of hemiparesis and an increase in body temperature (38.0–38.8 °C) immediately before reperfusion. Reperfusion was induced by withdrawing the filament from the origin of MCA after 1 h of occlusion under isoflurane anesthesia.

### Drug administration

FK506 was dissolved in 200 mg/mL polyoxyethylene hydrogenated castor oil 60 (HCO-60, Nikko Chemicals Co. Ltd., Tokyo, Japan) and ethanol, and then the solution was diluted by 10 fold with PBS. The final concentration of FK506 solution was 40 μg/mL. FK506 or FK506-liposomes (2 μmol phospholipid/rat) were administered intravenously at a dose of 100 μg/kg immediately after reperfusion. In the vehicle group, the solvent for dissolving FK506 was injected (0.5 mL/rat).

### Monitoring motor function

For evaluation of the motor functional outcome in t-MCAO rats, the treated rats underwent a 21-point neurological score analysis as described previously^31^. The measurement was performed at 1, 2, 3, 5, and 7 days after reperfusion. We had previously confirmed that normal and sham-operated (same procedure as MCAO surgery without filament insertion into the ICA) rats achieve 21 points in this test^14^.

### Measurement of cerebral blood flow

A skin incision was made on the head to expose the whole skull under isoflurane anesthesia as described above. To measure the cerebral blood flow, we performed a whole brain scan by using a laser-Doppler blood flowmeter (PeriScan PIM-III; Perimed AB, Stockholm, Sweden). The central position of the laser beam was set to lambda, and the scan range was set at 2.5 cm × 2.5 cm. Laser-Doppler perfusion imaging was conducted at 0, 12 h, and 1, 2, 3, 4, 5, 6, and 7 days after reperfusion. The ratio of the total cerebral blood flow in the ischemic brain hemisphere to that in the non-ischemic hemisphere was calculated.

### Brain damage assessment

To assess the brain cell damage, we dissected the brains of t-MCAO rats after 3 and 7 days of reperfusion and cut them into 2-mm thick coronal slices with a rat brain slicer (Muromachi Kikai, Tokyo, Japan). Then, the slices were incubated for 30 min at 37 °C in 2% TTC (Wako Pure Chemical Ind. Ltd.) solution to stain viable cells. The damaged brain volume (white area) was calculated with an image-analysis system (NIH Image J).

### Immunostaining for NeuN

FK506-liposomes (100 μg/kg FK506 equivalent, 2 μmol phospholipid/rat) or PBS were intravenously injected into t-MCAO rats immediately after reperfusion. Seven days after reperfusion, their brains were sliced into 2-mm coronal sections. Then, the slices were embedded in optimal cutting temperature compound (Sakura Finetek., Co. Ltd., Tokyo, Japan), and frozen in dry ice/ethanol. The frozen brains were cut into 10-μm sections, and immunostained for NeuN. In brief, the sections were incubated with 1% bovine serum albumin in PBS for 10 min at room temperature, and then incubated with Alexa fluor 488-conjugated anti-NeuN antibody (Millipore, Billerica, MA, USA) for 3 h at room temperature. Next, the sections were fixed in 4% paraformaldehyde for 10 min at room temperature. After adding DAPI solution (1.0 μg/mL, Molecular Probes, Eugene, OR, USA), the sections were mounted using Perma Fluor Aqueous Mounting Medium (Thermo Shandon, Pittsburgh, PA, USA). The fluorescence of the sections was observed by using an LSM 510 META microscope system (Carl Zeiss, Co., Ltd., Germany). For quantifying the number of neuronal cells, NeuN-positive cells were counted from each of 8 separate areas.

### PET imaging and autoradiography

The synthesis of [^18^F]BCPP-EF was performed as described previously^7^,^8^. t-MCAO rats were intravenously injected with FK506-liposomes (100 μg/kg FK506 dose, 2 μmol phospholipid/rat) or PBS immediately after reperfusion following 1 h of occlusion. Then, [^18^F]BCPP-EF (10 MBq/rat) was intravenously injected into the rats for PET scanning at 1, 3, and 7 days of reperfusion. Immediately after each injection, the PET scan for detecting MC-I activity was performed with a high-solution animal PET scanner (SHR-38000; Hamamatsu Photonics, Hamamatsu, Japan) for 60 min under anesthesia by an intraperitoneal injection of chloral hydrate (400 mg/kg, *i.p.*), followed by continuous infusion of it (100 mg/kg/h, *i.v.*) during the scanning. After each PET experiment, the rats were placed on an animal CT apparatus (Clairvivo CT; Shimadzu Corp, Japan) to obtain CT images. After the PET measurements at Day 7, the brain of the rat was sliced into 2-mm coronal sections, and the sections were set on an imaging plate for autoradiography using a bio-imaging analyzer system (FLA-7000, Fuji Film, Tokyo, Japan). The PET images from 10 to 30 min after the injection were reconstructed to obtain standardized uptake value (SUV) images. SUV is defined as the regional radioactivity concentration normalized by the total injected dose and body weight. To determine the volume of damaged brain from the PET images, volume of interests (VOIs) in the reconstructed PET images from 10 to 30 min after the start of PET scanning were calculated using PMOD software (PMOD Technologies Ltd, Zurich, Switzerland). VOIs were defined for damaged brain region in which SUV was less than 60% of average SUV in the corresponding non-ischemic side. We previously confirmed that there was no difference in the SUV of [^18^F]BCPP-EF between right and left brain hemispheres of normal rats^8^.

### Statistical analysis

The statistical differences in the results of monitoring motor function and measuring cerebral blood flow were assessed by two-way repeated-measures analysis of variance (ANOVA) followed by Tukey *post-hoc* test. In other experiments, the statistical differences in more than 3 groups were assessed by one-way ANOVA followed by Tukey *post-hoc* test, whereas those in 2 groups were evaluated by using Student’s *t-test*. Data were presented as the mean ± S.D.

## Additional Information

**How to cite this article**: Fukuta, T. *et al*. Non-invasive evaluation of neuroprotective drug candidates for cerebral infarction by PET imaging of mitochondrial complex-I activity. *Sci. Rep.* **6**, 30127; doi: 10.1038/srep30127 (2016).
